# The association of HLA-C alleles with multiple myeloma in Chinese patients

**DOI:** 10.1186/s40164-018-0112-y

**Published:** 2018-08-23

**Authors:** Xiaojing Wang, Gang An, Jiying Wang, Yan Zhang, Qinghua Li, Hui wei, Lugui Qiu, Kun Ru

**Affiliations:** 1 Department of Pathology and Lab Medicine, Institute of Hematology and Blood Diseases Hospital, Chinese Academy of Medical Sciences and Peking Union Medical College, Tianjin, 300020 China; 2 Department of Lymphoma, Institute of Hematology and Blood Diseases Hospital, Chinese Academy of Medical Sciences and Peking Union Medical College, Tianjin, China; 3 Department of Leukemia, Institute of Hematology and Blood Diseases Hospital, Chinese Academy of Medical Sciences and Peking Union Medical College, Tianjin, China

**Keywords:** Multiple myeloma, Human leukocyte antigen, Allele, Chinese population

## Abstract

**Background:**

The positive association of multiple myeloma (MM) risk with HLA-C loci C*07:02 g and C*02:02 g, and the negative association of that with C*05:01 g were statistically significant in Whites have recently been reported. However, no association between HLA-C alleles and MM risk was found in Asians/Pacific Islanders. Here we identified 316 Chinese patients with MM, and reported the results of our investigation of HLA-C in MM in Chinese population.

**Methods:**

We identified 316 Chinese patients with MM diagnosed in our hospital, and typed for HLA-C by using Sanger sequence-based typing. The control was from laboratories of China Marrow Donor Program (CMDP), where HLA high resolution was provided in 564,856 volunteer adult donors.

**Results:**

In contrast to the association of MM risk in Whites, we did not find the similar association in Chinese population. Nevertheless, four new associations between the MM risk were identified in Chinese patients. Our data demonstrated that Chinese patients with MM carry significantly increased frequencies of HLA-C*03:03 (FDR = 0.0269), HLA-C*07:63 (FDR = 0.0278) and HLA-C*08:22 (FDR = 0.0442) comparing with controls, while significantly decreased frequency of HLA-C*01:02 (FDR = 0.0414) comparing with controls.

**Conclusion:**

Therefore, HLA-C region is a key risk locus for MM in Chinese population.

## Background

Genetic predisposition studies in patients with hematological neoplasm include a large number of significant associations with the human leukocyte antigen (HLA) region. Niens et al. provided evidence that HLA-A*01:01 was associated with an increased and A*02:01 with a decreased risk of EBV^+^ Hodgkin lymphoma [1]. Alcoceba et al. found diffuse large B cell lymphoma (DLBCL) patients had a higher phenotypic frequency of HLA-DRB1*01 and a lower frequency of HLA-C*03 compared with healthy individuals [2]. Our previous studies showed that several HLA alleles were associated with susceptibility in leukemia [3, 4].

Multiple myeloma (MM) is a malignancy of plasma cells. Beksac et al. described that the positive association of MM risk with HLA-C loci C*07:02 g and C*02:02 g, and the negative association of that with C*05:01 g were statistically significant in Whites. However, among 74 Asian/Pacific Islander patients with MM and 50,000 controls, no association between HLA-C alleles and MM risk was found [5]. The previous study about MM association with HLA in Asian/Pacific Islander patients are limited by sample size. In order to determine whether or not HLA-C is playing a role, in this article, we expand the study samples and report the results of our investigation of HLA-C in MM in Chinese population.

## Methods

### Patients

From the medical record in last 5 years, we identified 316 Chinese patients with MM diagnosed in our hospital, including 200 males (63.3%) and 116 females (36.7%). The median age was 60 years (range 26–81 years).

### HLA-C high resolution typing

Genomic DNAs of patients’ peripheral blood were extracted using QIAamp DNA Blood Mini Kit (Qiagen, Hilden, Germany). Patients were typed for HLA-C by using Sanger sequence-based typing (GenDx, Netherlands). The sequence products were separated on ABI 3730 Genetic Analyzer (Hitachi High-Technologies Corporation, Tokyo, Japan), and analyzed with SBTengine analysis software (GenDx, Netherlands). Ambiguous allele combinations were solved by GSSP (Group Specific Sequencing Products) primers (GenDx, Netherlands).

In addition, public data were used as control from laboratories of China Marrow Donor Program (CMDP), where HLA high resolution was provided in 564,856 volunteer adult donors using DNA-based methods (Sanger sequence-based typing or sequence-specific oligonucleotide) [6].

### Statistical analysis

Allele frequency (AF) is the frequency (proportion) of alleles in a population sample (n), so AF = Alleles/2n.

By using Chi square and Fisher exact tests, comparisons between HLA-C alleles of MM patients and the controls were performed. SPSS 17.0 for Windows was used for analysis and *P *< 0.05 was considered to be statistically significant. Odds ratio (OR) and 95% confidence interval (CI) were determined. The *P*-values were adjusted for multiple testing using false discovery rate (FDR) with a 5% threshold [7].

## Results

The allele frequencies, i.e. gene frequencies, instead of phenotype frequencies were examined as recommended for HLA-disease association studies [8]. As listed in Table 1, there were 31 HLA-C alleles in 316 patients with MM. After Chi square and Fisher exact tests, we found nine alleles had significant *P*-values. The results indicated that the frequencies of C*01:02 (11.8671% vs. 15.8875%, OR = 0.713), C*03:02 (3.9557% vs. 5.93195%, OR = 0.652), C*07:02 (12.3418% vs. 15.19139%, OR = 0.786) were decreased, whereas the frequencies of C*03:03 (9.9684% vs. 6.93407%, OR = 1.486), C*06:02 (11.5506% vs. 8.85456%, OR = 1.344), C*07:63 (0.3165% vs. 0.00938%, OR = 33.831), C*08:22 (2.0570% vs. 0.87066%, OR = 2.391), C*12:02 (4.5886% vs. 3.13204%, OR = 1.487), C*17:01 (0.4747% vs. 0.10215%, OR = 4.664) were increased in Chinese patients with MM.

**Table 1:** Allele frequencies of HLA-C alleles in patients with MM and in controls

HLA-C	MM (n = 316)	Control (n = 564,856)	*P*-value	OR	95% CI	FDR *P*-value
C*01:02	75 (11.8671%)	179,483 (15.8875%)	0.005706	0.713	0.560–0.907	*0.0442*
C*01:03	4 (0.6329%)	7098 (0.6283%)	1.000000	1.007	0.377–2.693	1.0333
C*01:06	1 (0.1582%)	959 (0.08489%)	0.415580	1.865	0.262–13.276	0.6135
C*02:02	5 (0.7911%)	7796 (0.69009%)	0.947008	1.148	0.476–2.768	1.0873
C*03:02	25 (3.9557%)	67,104 (5.93195%)	0.034862	0.652	0.437–0.973	0.1544
C*03:03	63 (9.9684%)	78,335 (6.93407%)	0.002686	1.486	1.145–1.928	*0.0278*
C*03:04	54 (8.5443%)	112,096 (9.92253%)	0.246598	0.848	0.642–1.121	0.4497
C*04:01	35 (5.5380%)	65,172 (5.7689%)	0.803415	0.958	0.681–1.347	1.0377
C*04:03	4 (0.6329%)	11,488 (1.0169%)	0.336047	0.620	0.232–1.657	0.5209
C*04:82	2 (0.3165%)	2771 (0.24528%)	1.000000	1.291	0.322–5.176	1.0000
C*05:01	2 (0.3165%)	9626 (0.85208%)	0.142966	0.369	0.092–1.480	0.3409
C*06:02	73 (11.5506%)	100,031 (8.85456%)	0.017080	1.344	1.053–1.716	0.1059
C*07:01	7 (1.1076%)	6394 (0.56598%)	0.121410	1.968	0.934–4.146	0.3136
C*07:02	78 (12.3418%)	171,619 (15.19139%)	0.046002	0.786	0.620–0.996	0.1585
C*07:04	6 (0.9494%)	9810 (0.86836%)	0.826323	1.094	0.490–2.445	1.0246
C*07:06	8 (1.2658%)	9024 (0.79879%)	0.187375	1.592	0.792–3.199	0.4149
C*07:154	1 (0.1582%)	154 (0.01363%)	0.083042	11.624	1.625–83.175	0.2340
C*07:63	2 (0.3165%)	106 (0.00938%)	0.001734	33.831	8.333–137.341	*0.0269*
C*08:01	54 (8.5443)	96,496 (8.54165%)	0.998093	1.000	0.757–1.322	1.0669
C*08:02	5 (0.7911%)	3406 (0.30149%)	0.059990	2.637	1.093–6.362	0.1860
C*08:03	8 (1.2658%)	8754 (0.77489%)	0.237989	1.642	0.817–3.298	0.4611
C*08:22	13 (2.0570%)	9836 (0.87066%)	0.001336	2.391	1.380–4.143	*0.0414*
C*12:02	29 (4.5886%)	35,383 (3.13204%)	0.035605	1.487	1.025–2.159	0.1380
C*12:03	16 (2.5316%)	21,799 (1.92961%)	0.271411	1.320	0.803–2.169	0.4674
C*14:02	23 (3.6392%)	48,357 (4.28047%)	0.425915	0.845	0.557–1.281	0.6002
C*14:03	9 (1.4241%)	11,482 (1.01637%)	0.307052	1.407	0.728–2.717	0.5010
C*15:02	21 (3.3228%)	37,813 (3.34714%)	0.972856	0.992	0.642–1.534	1.0771
C*15:04	1 (0.1582%)	432 (0.03824%)	0.215112	4.143	0.581–29.522	0.4446
C*15:05	3 (0.4747%)	7914 (0.70053%)	0.658438	0.676	0.217–2.102	0.8875
C*16:02	2 (0.3165%)	2292 (0.20288%)	0.847597	1.562	0.389–6.261	1.0106
C*17:01	3 (0.4747%)	1154 (0.10215%)	0.027885	4.664	1.498–14.522	0.1441

Italic values indicate FDR < 0.05

*OR* odds ratio, *CI* confidence interval, *FDR* false discovery rate

The *P*-values were adjusted for multiple testing using FDR. Further analysis among those nine alleles revealed that the adjusted *P*-values from C*01:02, C*03:03, C*07:63 and C*08:22 alleles were < 0.05 after adjustment for multiple comparisons. Therefore, our data demonstrated that Chinese patients with MM carry significantly increased frequencies of HLA-C*03:03 (FDR = 0.0269), HLA-C*07:63 (FDR = 0.0278) and HLA-C*08:22 (FDR = 0.0442) comparing with controls, while significantly decreased frequency of HLA-C*01:02 (FDR = 0.0414) comparing with controls.

## Discussion

The HLA system, which is located on 6p21.3, is the most polymorphic of all human genetic systems. Some HLA alleles occur at a much higher frequency in those suffering from certain diseases than in the general population. In genome-wide association study (GWAS) demonstrate that the HLA region is a key risk locus for mature B cell malignancies [2, 9]. Europe PMC Funders Group conducted a GWAS study and identified a risk variant within HLA region for MM risk, but the associated single-nucleotide polymorphisms (SNPs) at 6p21.33 (rs2285803) was not directly characterized with classical HLA typing [10].

In our study we explored the association of HLA-C alleles with MM in Chinese patients. In contrast to the positive association of MM risk with C*07:02 g and C*02:02 g, as well as the negative association of that with C*05:01 g in Caucasian population, we failed to find the similar association in Chinese population. Nevertheless, some new associations between the MM risk and HLA-C alleles were identified in Chinese patients. It appears that the association of MM risk and HLA alleles varies depending on different ethnic groups [5].

Multiple comparisons correction refers to the need to correct a significance level for the number of hypothesis tests performed. One of the most widely used multiple testing criterions for controlling errors of false discoveries is False discovery rate (FDR). Therefore, our *P*-values were adjusted for multiple testing using FDR. Our results indicated that the positive association of MM risk with C*03:03, C*07:63 and C*08:22, as well as the negative association of that with C*01:02 in Chinese population.

The frequency distribution of HLA alleles differs significantly among different human populations [11]. According to the Chinese common and well-documented (CWD) 2.2 catalogue, which was assembled by CMDP in February 2016, C*01:02, C*03:03, C*08:22 are common alleles (gene frequencies greater than 0.1% [12]), and C*07:63 is a well-documented allele (alleles observed in at least three independent unrelated individuals [12]) in Chinese population. However, based on the CWD 2.0.0 catalogue assembled from worldwide population in 2012 by American Society for Histocompatibility and Immunogenetics (ASHI), C*01:02, C*03:03 are common alleles, and C*08:22, C*07:63 are rare alleles in other ethnic populations [13]. This explains why the true association of C*08:22 and C*07:63 alleles with MM were not discovered among Whites and Blacks.

Besides MM, many previous studies showed that HLAs were associated with other haematological cancers. An association study found 6 protective or predisposing HLA-C alleles for chronic lymphocytic leukemia (CLL) in whites. Three alleles were protective (C*05:01, C*07:01, C*16:01) and 3 predisposing (C*03:04, C*12:03, C*16:02) [14]. A US population-based case–control study showed that HLA-B*08 was independently associated with non-Hodgkin lymphoma (NHL) risk in whites [15]. In Chinese population, our previous results indicated that as compared with the control, the frequency of HLA-DRB1*09 in ALL group significantly decreased (10.87% versus 16.08%, *P *= 0.014), while the frequency of HLA-B*18 in CML group was significantly higher (1.28% versus 0.20%, *P *= 0.039) [3]. We also found the frequency of HLA-DRB1*15 in childhood ALL was higher than those in control (22.62% versus 16.81%, *P *= 0.018) [4].

In summary, we discovered the positive association of MM risk with C*03:03, C*07:63 and C*08:22, as well as the negative association of that with C*01:02 in Chinese population. Therefore, HLA-C region is a key risk locus for MM in Chinese population.
